# Awareness and perceptions of systemic inflammation in atherosclerotic cardiovascular disease and chronic kidney disease: The SPARK-CVD China survey

**DOI:** 10.1016/j.athplu.2026.100570

**Published:** 2026-06-06

**Authors:** Xiaoxia Liu, Yuanlin Guo, Ban Zhao, Haobo Teng, Wenyan Liu, Kun Xiao, Changsheng Ma

**Affiliations:** a Department of Cardiology, Beijing Anzhen Hospital, Capital Medical University, Beijing, China; b Cardiometabolic Medicine Center, Fuwai Hospital, Chinese Academy of Medical Sciences and Peking Union Medical College, Beijing, China; c Department of Nephrology, Beijing Hospital, National Center of Gerontology, Institute of Geriatric Medicine, Chinese Academy of Medical Sciences, Beijing, China; d Novo Nordisk (Shanghai) Pharma Trading Co., Ltd., Beijing, China

**Keywords:** ASCVD, CKD, Systemic inflammation, High-sensitivity C-Reactive protein

## Abstract

**Background and aims:**

Cardiovascular disease (CVD) remains a leading cause of death in China. Systemic inflammation (SI) is an emerging risk factor in atherosclerotic CVD (ASCVD) and chronic kidney disease (CKD). High-sensitivity C-reactive protein (hsCRP) is increasingly recognised for prognostication. The SPARK-CVD China survey assessed Chinese cardiologists' and nephrologists’ awareness and perceptions of SI and hsCRP in patients with both ASCVD and CKD.

**Methods:**

A nationwide cross-sectional survey was conducted (September to December 2024) across 31 provinces in China mainland among physicians with ≥3 years of clinical experience and managing ≥20 adult patients with both ASCVD and CKD per month. Descriptive and comparative statistics were used.

**Results:**

Among 1500 respondents, SI was used more to aid treatment than diagnosis (65.2% vs 45.5%). Although 73.3% viewed SI as a key determinant of cardiovascular events, only 35.2% discussed SI as a risk factor with patients. Non-testers cited no expected impact on decisions (71.3%), lack of guideline direction (44.0%), and limited treatments (37.2%). A knowledge–practice gap for hsCRP was observed: 29.7% identified hsCRP unprompted versus 87.7% when prompted; perceived diagnostic thresholds varied widely. Fewer than 1/4 of ASCVD and/or CKD patients would be prescribed colchicine; barriers included limited experience (55.2%), potential contraindications (54.1%), and side effects (47.1%). Cardiorenal benefits of GLP-1 receptor agonists were widely recognised (97.9%), with 76.5% attributing benefits partly to anti-inflammatory effects.

**Conclusion:**

SI is acknowledged but inconsistently operationalised domestically. Targeted professional education, explicit guideline recommendations, and further evidence for risk-stratified, inflammation-guided care may help refine treatment pathways for ASCVD with CKD.

## Highlights

• 70%+ of physicians saw systemic inflammation (SI) as a determinant of CV events, less than 1/3 discussed SI with patients.
• Less than half of physicians tested SI at diagnosis, while over half did so while treating patient with ASCVD and CKD.
• Non-testers cited lack of impact on decisions, lack of guideline direction, and limited treatments for SI as main reasons.
• Less than 1/3 mentioned high-sensitivity C-reactive protein unaided indicating low perceived importance of SI testing.
• The largest importance-satisfaction gap regarding SI testing was found regarding availability of SI-targeted treatment.

## Graphical abstract

Awareness and perceptions of SI in ASCVD and CKD.

**Figure:**
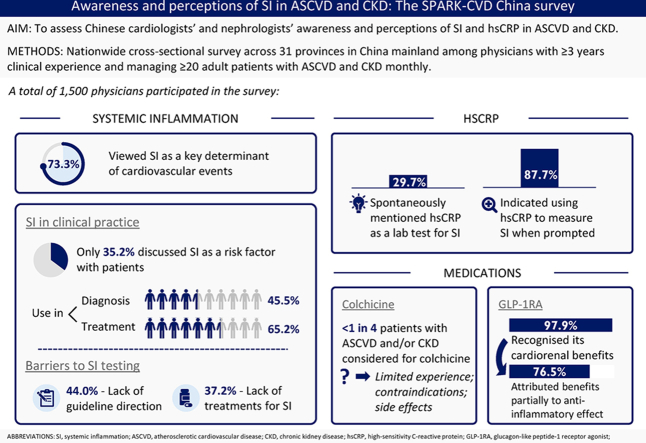

## 1 Introduction

Despite advanced evidence-based treatments, cardiovascular disease (CVD) and chronic kidney disease (CKD) are among the most prevalent non-communicable diseases and contributors to death and disease burden in China and globally [1,2]. Atherosclerotic cardiovascular disease (ASCVD) contributes to significant burden in China, with 13.8% of the adult population classified as high 10-year risk and CVD accounting for over 40% of deaths in 2019 [3,4]. The estimated prevalence of CKD has notably increased from 6.7% (1990) to 10.6% (2019), along with a corresponding increase in mortality from 8.3 to 13.8 per 100,000 [5]. Residual risk of CVD remains inadequately addressed by current standard-of-care therapies, highlighting a warranted imperative to discover and evaluate next-generation molecular targets for cardiovascular protection.

Systemic inflammation (SI) is increasingly recognised as an important risk factor that significantly contributes to the development and progression of ASCVD and CKD [[6], [7], [8], [9]]. This association has been endorsed by some guidelines, which recommend considering anti-inflammatory treatments for patients with ASCVD [10,11]. High-sensitivity C-reactive protein (hsCRP) is a relatively established, routinely employed inflammatory biomarker that serves as an independent predictor and incremental tool for cardiovascular risk prediction and risk stratification in both primary and secondary prevention settings [11]. However, current real-world clinical practice patterns and guideline recommendations remain inconsistent [12,13]. Furthermore, given the limited literature regarding SI and hsCRP in the context of patients with ASCVD and CKD in China, the clinical awareness and perceptions towards role of SI and hsCRP as a biomarker among Chinese physicians are not well established.

Colchicine is increasingly recognised to be a promising option in anti-inflammatory therapy [[14], [15], [16]]. Colchicine inhibits tubulin polymerisation, thereby suppressing NLRP3 inflammasome activation and primarily reducing IL-1β production, with decreases in IL-6 and hsCRP occurring secondarily as downstream effects of its broader anti-inflammatory action [15]. It has demonstrated its efficacy in lowering recurrent ASCVD events, resulting in a conditional class IIa recommendation from the European Society of Cardiology (ESC) for high-risk chronic coronary patients [[14], [15], [16]].

Glucagon-like peptide-1 receptor agonist (GLP-1RA) is a novel drug class that is currently prescribed and primarily used in the management of type 2 diabetes and obesity [17]. Though not prescribed as anti-inflammatory agents, GLP-1RAs exert anti-inflammatory mechanisms in ASCVD that may contribute to cardiorenal protection [[18], [19], [20]]. Preclinical studies suggest that GLP-1RAs possess anti-inflammatory and renal benefits, which contributes to mitigating endothelial dysfunction and stabilise atherosclerotic plaques, thereby contributing to the observed reduction in cardiovascular risk in clinical trials, beyond their effects on glucose control and weight reduction [19,20].

The SPARK-CVD China study was a real-world, cross-sectional survey designed to evaluate the awareness and perceptions of Chinese cardiologists and nephrologists regarding the role of SI and hsCRP as a biomarker, and the management patterns in patients with both ASCVD and CKD. Specifically, the study aims to address several key objectives: firstly, to understand the level of awareness and perceptions among these physicians about the role of SI in patients with both ASCVD and CKD within real-world clinical settings; secondly, to assess the awareness and perceptions about the role of hsCRP as a biomarker for identifying and managing SI in these patients; and thirdly, to investigate their awareness and perceptions of the anti-inflammatory effect of colchicine and GLP-1RA in patients with ASCVD and/or CKD. By addressing these objectives, the findings from this study could fill existing knowledge gaps regarding physician awareness and perceptions towards SI and could be further utilised to enhance clinical attention related to the management of SI in Chinese patients with both ASCVD and CKD.

## 2 Methods

### 2.1 Study design

SPARK-CVD China was a cross-sectional, observational, descriptive, and survey-based study conducted from 9 September to 5 December 2024. Cardiologists and nephrologists from tertiary and secondary hospitals across 31 provinces in China mainland (n = 1500) were eligible for the analysis. The sample size of 1500 is selected to ensure the precision of the study with sampling error of 2.53%.

### 2.2 Variables

The primary outcomes were physician's awareness and perceptions towards the role of SI in the identification, treatment and management of patients with ASCVD and CKD measured by one or a combination of single-select or multi-select answers from a defined list, numerical answers, and 7-point Likert scales.

### 2.3 Bias

Participation was limited to respondents who met the inclusion criteria and fully completed the survey. Voluntary participation and the source of physician respondents may have constituted selection bias, which is inherent to survey-based studies.

However, bias was minimised where possible by not specifying the study topic in invitations and using general framing (e.g., a survey about cardiovascular disease); carefully designing the screener so respondents were unaware of the study purpose before meeting eligibility criteria; applying broad screening criteria to ensure a diverse physician sample representative of the average case; employing a wide geographical coverage to partially attenuated selection bias; using neutral wording to avoid leading to responses; and rotating the display order of response options.

### 2.4 Participants

Eligible cardiologists and nephrologists should be practising in either in tertiary or secondary hospitals, having at least 3 years of consultant-level clinical experience, and managing a minimum of 20 adult patients with ASCVD and CKD monthly.

### 2.5 Ethical procedures

The study was conducted in accordance with the Declaration of Helsinki. The study protocol, informed consent form (ICF) and study questionnaire were submitted, reviewed and approved independently by the Beijing Anzhen Hospital Medical Ethics Committee. Informed consent was obtained from each physician respondent.

### 2.6 Steering committee

A steering committee comprising 3 senior consultant board-registered cardiologists and 1 senior consultant board-registered nephrologist was established to provide direction on the study and endorse the findings of the research.

### 2.7 Data sources

Participants entered responses directly into a secure online survey platform. Survey data were exported in a uniform format to the statistical analysis environment and to the study archive. No data from qualified responses were truncated or discarded; all analyses were performed on the original dataset.

### 2.8 Missing data

The survey was implemented with skip logic and gated response options to ensure each question targeted the relevant respondents. The survey programme was also thoroughly tested before launch. Where appropriate, a “don't know/not sure” option was provided for respondents unable to provide accurate answers. No unintended missing values were observed. During analysis, the denominator (i.e. the base) was appropriately adjusted to include non-missing data only to preserve data accuracy.

### 2.9 Statistical methods

SPSS Statistics (Version 28.0.1.1, IBM Corp.) and SAS (Version 9.4, SAS Institute Inc.) were used for analysis of survey datasets. Descriptive statistics (total and subgroup analysis) and inter-group significance testing (ANOVA, T-test, Chi-square, and Fisher's Exact Test) were conducted in SPSS. The *p*-value adjustments for multiple comparisons and correlations were implemented in SAS.

All data were analysed descriptively on awareness, perception and clinical management pattern variables. Continuous variables were presented as mean ± SD. Categorical variables were presented as percentages (%). Hypotheses testing used ANOVA and T-test for continuous variables, and Chi-square and Fisher's Exact Test for categorical variables. Correlation analysis was utilised to investigate the relationship between perceptions and behaviours. Significance level is set a two-sided α = 0.05.

## 3 Results

### 3.1 Baseline characteristics of physicians

Of the 1768 physicians who responded to the survey invitation, 1500 met the eligibility criteria, completed the survey and were included in the analysis. Physicians had a mean age of 44.0 ± 7.1 years and 54% male composition (Table 1). Distribution of physician titles were almost similar among cardiologists and nephrologists, with chief doctors at 21.5%, vice chief doctors at 45.0%, and attending doctors at 33.5%, overall. The mean duration of clinical practice was 18.2 ± 7.3 years overall and was slightly higher among cardiologists (18.4 ± 7.4 years) than nephrologists (17.4 ± 7.0 years). Physicians managed a mean of 278 ± 174 patients monthly. Cardiologists and nephrologists treated 40 ± 25 and 61 ± 44 patients with ASCVD and CKD, respectively.

**Table 1:** Participant demographics and ASCVD risk factors discussed with patients.

Respondent Demographics	Total	Cardiologists	Nephrologists	Tertiary	Secondary
Number of respondents	1500	1200	300	1060	440
Age in years, mean	44.0 ± 7.1	44.2 ± 7.3	43.5 ± 6.7	44.1 ± 7.1	43.9 ± 7.2
Gender, male%	54.0	56.2∗	44.8	54.1	53.6
Physician title, %					
Chief doctor	21.5	22.4	18.0	24.8∗	13.4
Vice chief doctor	45.0	44.8	45.7	46.2	42.0
Attending doctor	33.5	32.8	36.3	29.0	44.6∗
Years in practice, mean	18.2 ± 7.3	18.4 ± 7.4∗	17.4 ± 7.0	18.4 ± 7.4∗	17.6 ± 7.3
Patients per typical month, mean					
Total patients, any condition	278 ± 174	287 ± 176∗	243 ± 158	298 ± 178∗	230 ± 152
ASCVD	154 ± 106	170 ± 109∗	88 ± 59	166 ± 110∗	125 ± 89
Arrhythmia	55 ± 44	55 ± 44	-	59 ± 48∗	43 ± 28
Heart failure	51 ± 47	54 ± 50∗	37 ± 31	55 ± 51∗	41 ± 34
Category of ASCVD seen/treated monthly, mean					
Coronary heart disease	116 ± 86	131 ± 87∗	53 ± 38	125 ± 90∗	93 ± 71
Acute myocardial infarction	13 ± 14	15 ± 14∗	5 ± 6	14 ± 14∗	9 ± 10
Cerebrovascular disease	38 ± 30	39 ± 31∗	33 ± 27	40 ± 31∗	33 ± 27
Symptomatic peripheral artery disease	19 ± 18	21 ± 19∗	13 ± 14	21 ± 19∗	16 ± 15
Patients with ASCVD and CKD seen/treated monthly, mean	44 ± 31	40 ± 25	61 ± 44∗	47 ± 32∗	38 ± 28
**Risk factors of ASCVD discussed with patients at risk (n, %)**					
Hypertension	1441(96.1)	1157(96.4)	284(94.7)	1022(96.4)	419(95.2)
Hyperglycaemia	1363(90.9)	1081(90.1)	282(94.0)∗	963(90.9)	400(90.9)
Hyperlipidaemia	1357(90.5)	1087(90.6)	270(90.0)	963(90.9)	394(89.6)
Overweight or obesity	1267(84.5)	1011(84.3)	256(85.3)	916(86.4)∗	351(79.8)
Lifestyle habits (i.e., diet, exercise)	1254(83.6)	1010(84.2)	244(81.3)	892(84.2)	362(82.3)
Impacts of Tobacco use	1194(79.6)	968(80.7)∗	226(75.3)	847(79.9)	347(78.9)
CKD	1054(70.3)	787(65.6)	267(89.0)∗	747(70.5)	307(69.8)
Impacts of alcohol use	970(64.7)	778(64.8)	192(64.0)	669(63.1)	301(68.4)
Ageing	918(61.2)	744(62.0)	174(58.0)	655(61.8)	263(59.8)
Hyperuricemia	896(59.7)	671(55.9)	225(75.0)∗	629(59.3)	267(60.7)
Genetics/family history	853(56.9)	711(59.3)∗	142(47.3)	616(58.1)	237(53.9)
Sleep apnea	758(50.5)	651(54.3)∗	107(35.7)	557(52.6)∗	201(45.7)
Cardiac enzymes	572(38.1)	495(41.3)∗	77(25.7)	418(39.4)	154(35.0)
Systemic inflammation	528(35.2)	413(34.4)	115(38.3)	394(37.2)∗	134(30.5)
Side effects of a medication for another condition	385(25.7)	309(25.8)	76(25.3)	269(25.4)	116(26.4)
MASH^b^	326(21.7)	265(22.1)	61(20.3)	216(20.4)∗	110(25.0)
Rheumatoid arthritis	270(18.0)	227(18.9)	43(14.3)	200(18.9)	70(15.9)

^a.^ p < 0.05, denoted by “∗”.

^b^. MASH: metabolic dysfunction-associated steatohepatitis.

### 3.2 Perception on the role and clinical usage of SI in the management of ASCVD and CKD

**Fig. 1:**
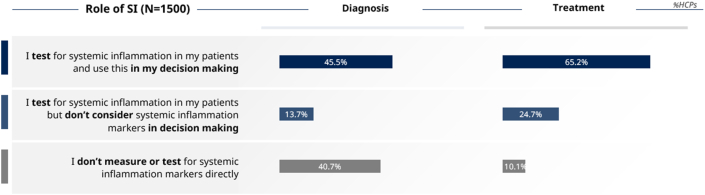
Role and usage of SI in the diagnosis and treatment of patients with ASCVD and CKD Role and usage of SI when physicians diagnose and when physicians treat patients with ASCVD and CKD (n = 1500).

When diagnosing patients with both ASCVD and CKD, 45.5% of physicians reported testing for SI and incorporating the results into decision-making. When it comes to treatment, this proportion increased to 65.2% (Fig. 1). However, 40.7% and 10.1% of physicians did not measure or test for SI during diagnosis and treatment, respectively.

**Fig. 2:**
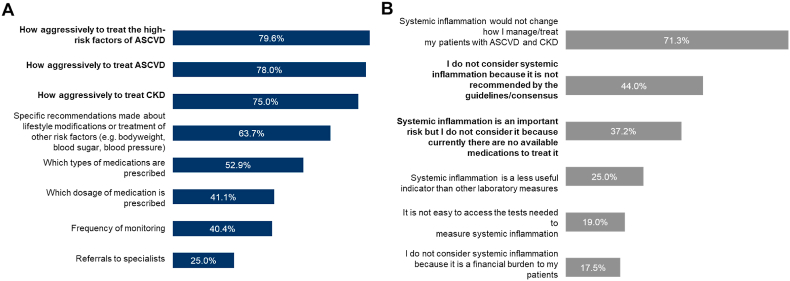
Influences of SI and reasons for not considering SI in the management of patients with ASCVD and CKD (A) Influences of SI in the management of patients with ASCVD and CKD among physicians who claimed testing and using SI results in their treatment (n = 978); (B) Reasons for not considering SI in the management of patients with ASCVD and CKD (n = 457).

Among physicians who tested for SI (n = 978), more than three-quarters reported SI had influence on their clinical practice on management of ASCVD risk factors (79.6%), ASCVD (78.0%) and CKD (75.0%) (Fig. 2A). These findings aligned with physician attitudes towards SI where 73.3% considered SI a key determinant of cardiovascular events in patients with ASCVD and CKD (Supplementary Fig. 1), and 70% acknowledged its role in disease progression and prognosis. However, only 35.2% of physicians discussed SI as an ASCVD risk factor with their patients (Table 1).

Conversely, among physicians who did not consider SI testing (n = 457), most (71.3%) believed it would not alter their management strategies for ASCVD and CKD. Additionally, a lack of guideline recommendations (44.0%) and unavailable treatments (37.2%) were cited as top reasons for not considering SI testing (Fig. 2B). When we consider unmet needs of managing patients with ASCVD and CKD, the limited awareness of the role of SI in ASCVD (42.3%), the lack of effective treatment options for SI (34.4%) and limited biomarkers or tools to assess SI (30.6%) were key unmet needs reported (Supplementary Fig. 2).

In practice, monthly assessment of SI among patients with ASCVD and CKD was most reported (39.6%), followed by quarterly assessment (18.8%) (Supplementary Fig. 3).

### 3.3 hsCRP testing for assessing SI in patients with ASCVD and CKD

**Fig. 3:**
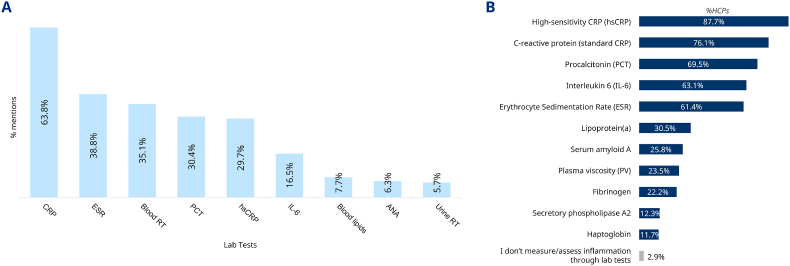
Unaided and aided awareness of SI lab tests (A) Unaided awareness: spontaneous/unprompted mentions of lab tests for SI (n = 1500); (B) Aided awareness: prompted mentions from a predefined list of SI lab tests (n = 1500).

Overall, less than a third (29.7%) mentioned hsCRP when asked to spontaneously list up to five lab tests for SI (Fig. 3A). In contrast, a majority (87.7%) indicated using hsCRP tests for SI when given predefined options (Fig. 3B). Regarding hsCRP thresholds, almost a third (31.3%) selected the value in the range of ≥2 and <3 mg/L while almost a fifth (17.3%) were unsure (Supplementary Fig. 4).

**Fig. 4:**
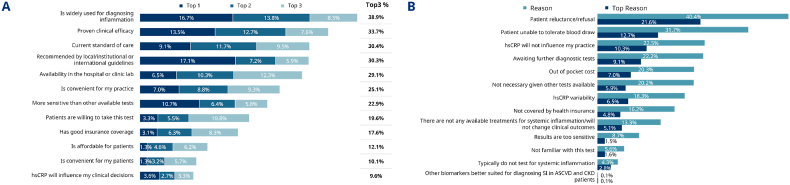
Reasons for and reasons for not considering hsCRP testing to assess SI in patients with ASCVD and CKD (A) Top 1-3 reasons for considering hsCRP testing to assess SI in patients with ASCVD and CKD (n = 1500); (B) Reasons and top reason for not considering hsCRP testing to assess SI in patients with ASCVD and CKD (n = 1500).

Fig. 4 illustrated the reasons for using and not using hsCRP testing for assessing SI in patients with ASCVD and CKD. Top reasons for use were its widespread use in diagnosing inflammation (38.9%), proven clinical efficacy for management (33.7%) and considering hsCRP testing to be standard of care (30.4%) (Fig. 4A). Conversely, top reasons against use were patient reluctance/refusal (40.4%, with 21.6% cited as the top reason), patients being unable to tolerate blood draws (31.7%, with 12.7% cited as top reason) and the perception that hsCRP testing would not influence the clinical practice (22.5%, with 10.3% cited as the top reason) (Fig. 4B). In importance–satisfaction ratings on a 10-point scale, proven clinical efficacy and test availability were rated most important (8.3 and 8.3), followed by guideline endorsement (8.2); and satisfaction was highest for test availability (8.1). The largest gap was for the availability of SI-specific treatments (importance 7.9 vs satisfaction 6.8) (Supplementary Fig. 5).

### 3.4 Perceptions of potential treatments for SI in ASCVD and CKD

Assuming the availability of colchicine, physicians would prescribe this medication to 22.1% of patients with both ASCVD and CKD, to 20.8% of patients with ASCVD only, and to 15.2% of patients with CKD only (Supplementary Fig. 6A). Key barriers towards prescribing colchicine to all CKD patients included limited experience with the medication (55.2%), potential contraindications (54.1%), and side effects such as diarrhoea (47.1%) (Supplementary Fig. 6B).

On the awareness and perceptions of GLP-1RA usage in patients with ASCVD and CKD, 97.9% of physicians recognised GLP-1RA's cardiorenal benefits (Supplementary Fig. 6C), of which 76.5% recognised the cardiorenal benefits being partially due to its anti-inflammatory effect (Supplementary Fig. 6D). A small minority (3.0%) that did not recognise the benefits of GLP-1RA perceived the limited evidence of direct anti-inflammatory effects of GLP-1RA (75.0%) and cardiorenal benefits of GLP-1RA were mainly driven by improvements in other cardiometabolic risk factors (70.5%) (Supplementary Fig. 6E).

## 4 Discussion

As the first nationwide study to characterise Chinese cardiologists' and nephrologists’ perceptions of SI in patients with both ASCVD and CKD, the SPARK-CVD survey provides a current view of how SI is recognised and managed in this specific patient group, while highlighting a disconnect between knowledge, perceptions in real-world practice. Three key observations emerged from this study on SI, hsCRP, and emerging anti-inflammatory therapies. Our study revealed a profound “knowledge and practice” gap among the physicians. Most respondents agreed that SI acted as a key driver in ASCVD and CKD development and progression, yet only one-third translated this belief into conversations with patients, and 40% ordered no inflammatory markers during the study period. These findings underscore that, unlike traditional risk factors such as low-density lipoprotein cholesterol, SI has yet to receive comparable attention in clinical practice and highlight the need to translate SI-guided strategies in routine care. Notably, there is an increased level of engagement in SI testing in the treatment stage, as evidenced by the 65.2% who both tested and utilised SI results, as compared to 45.5% for diagnosis. Besides, physicians who chose to measure SI during treatment regard its results as crucial for the routine management of patients with both ASCVD and CKD. Additionally, among physicians not considering SI in clinical decision-making, the principal reasons cited were the absence of explicit guideline recommendations and the lack of accessible targeted anti-inflammatory therapies. Collectively, these findings echoed prior multi-national FLAME-ASCVD study [21], where intensifying physician education, advancing guideline endorsement of cardiovascular anti-inflammatory strategies, and accelerating the development of targeted anti-inflammatory agents represent key approaches to addressing currently unmet clinical needs.

From specialists’ perspective, cardiologists and nephrologists share similar attitudes on SI but prioritised disease-specific outcomes (e.g., CVD vs. renal endpoints). For patients with ASCVD and CKD, there is a warranted need for therapies that can concurrently improve both cardiovascular and renal outcomes.

Regarding the use of hsCRP testing as a biomarker of SI, our research revealed gaps and variability in both perception and clinical application, where testing is employed reactively rather than proactively. From the survey, most physicians were aware of hsCRP when prompted, less than a third were spontaneously able to indicate hsCRP (87.7% vs. 29.7%), highlighting the disconnect between recognition and routine consideration. Proactive use of hsCRP to identify SI remains modest in clinical practice; Reasons for using hsCRP were disparate among physicians as no single reason was selected by over 40%, even among well-informed physicians. This is despite consistent evidence that persistently elevated hsCRP predicts cardiovascular risk in both primary and secondary prevention and guideline recommendations to treat hsCRP ≥2 mg/L as a risk-enhancing factor [22,23]. In China, given the high ASCVD burden and the test's low cost and accessibility, increasing hsCRP uptake may be a practical measure for screening out patients with SI risk that warrant increased attention. Although the majority accepted an hsCRP threshold value between 2 and 3 mg/L, nearly one-fifth remained uncertain about the exact value. Therefore, this lack of consensus underscores the need for enhancing physician understanding and optimising risk stratification. Furthermore, importance–satisfaction analyses revealed that the availability of tests and evidence for clinical efficacy were critical, with a significant gap noted in the lack of SI-specific treatments. This result echoes our earlier findings and once again underscores the current clinical demand for SI-specific targeted therapies.

Third, on physician perception of current anti-inflammatory strategies, attitude towards low-dose colchicine was cautious with less than one in four patients being considered for use on average due to concerns with limited clinical experience and potential contraindications in CKD. Although randomised trials have demonstrated cardiovascular risk reduction with low-dose colchicine in chronic and post-acute coronary syndromes [14,15], subsequent data have been mixed in specific subgroups, and concerns persisted regarding drug–drug interactions and contraindications in patients with renal impairment. GLP-1RAs have demonstrated anti-inflammatory effects in pre-clinical and clinical studies [20]. In our survey, 97.9% recognised its cardiorenal benefits and 76.5% of these recognised its cardiorenal benefits were partly due to anti-inflammatory effects. It should be emphasised that these cardiorenal indications are established based on dedicated cardiovascular and renal outcome trials [[24], [25], [26], [27]], and anti-inflammatory effects, while a potential mechanistic contributor, do not constitute a criterion for clinical selection. Given their established cardiorenal benefits, GLP-1RA may be considered as supplementary options to standard of care for ASCVD and CKD. However, further research is needed to discover more SI-targeted treatments, define optimal dosing and timing, delineate mechanisms of benefit, and identify patients most likely to benefit from targeted, safe cardiovascular anti-inflammatory therapies. These efforts could assist in updating traditional practices and integrating more comprehensive, guideline-supported approaches within the healthcare system in China.

### 4.1 Strengths & limitations

The SPARK-CVD study has several strengths that provide value towards ASCVD and CKD management. First, the questionnaire was designed to probe both cognition and practice: it distinguished diagnostic from treatment use of SI, combined spontaneous and prompted responses for hsCRP (thereby differentiating top-of-mind awareness from aided recognition), and included importance–satisfaction ratings. This multifaceted approach provides a granular picture of how SI is understood, measured, and acted upon in routine care. Second, the sampling strategy enhances external validity within China. This national, real-world survey included cardiologists and nephrologists across all 31 provinces and municipalities and spanned secondary and tertiary hospitals with minimal inclusion/exclusion criteria, enabling direct comparisons of perceptions and operational barriers across specialties, geographies, hospital tiers, and care settings, and offering a nuanced picture of the cardiorenal interface rarely captured in single-specialty or single-tier samples. Third, the survey simultaneously examined biomarker use (hsCRP) and therapeutic attitudes (colchicine, GLP-1RAs), linking perceptions of SI to concrete management choices and revealing implementation levers relevant to preventive practice. Finally, to our knowledge, this is the first national survey to characterise how Chinese physicians perceive and operationalise SI in patients with ASCVD and CKD, thereby adding context-specific evidence to an area of growing international interest.

On limitations, the reliance on self-administered online surveys, potentially skewing results towards younger demographics and affecting the findings' generalisability. The voluntary nature of participation could introduce recall bias, impacting the results' reliability. Additionally, as the questionnaire contained over 30 questions with an expected completion time of 30 min, response fatigue may have impacted the accuracy and validity of responses received [28]. Furthermore, the study was exclusively conducted in China mainland, which may limit its generalisability in clinical settings outside of China.

In conclusion, the SPARK-CVD China study provides a nationally representative overview of physician perceptions and practices regarding SI and current anti-inflammatory therapies in patients with both ASCVD and CKD. The study underscores a widespread recognition of SI's role among physicians managing ASCVD and CKD yet reveals gaps between awareness and practical implementation of SI testing in clinical settings in China. In the future, enhancing physician education, refining clinical guidelines to incorporate hsCRP testing could be important steps toward refining and informing care strategies. To address the unmet therapeutic gap in SI, research should focus on developing, validating, and implementing SI-targeted treatment that are both safe and effective for routine clinical use.

## Data availability statement

The datasets generated during and/or analysed during the current study are available from the corresponding author on reasonable request.

## Author contributions

All authors were responsible for the survey study concept and survey design, data collection, statistical analysis plan. All authors contributed to the data analysis and interpretation, drafted and critically reviewed the manuscript, gave final approval of the version to be published, and agree to be accountable for all aspects of the work.

## Financial support

This study was funded by 10.13039/501100004191Novo Nordisk (Shanghai) Pharma Trading Co., Ltd., Beijing, China.

## Declaration of competing interest

The authors declare the following financial interests/personal relationships which may be considered as potential competing interests: Haobo Teng, Wenyan Liu reports a relationship with Novo Nordisk Shanghai Pharmaceutical Trading Co Ltd that includes: employment and equity or stocks. Kun Xiao reports a relationship with Novo Nordisk Shanghai Pharmaceutical Trading Co Ltd that includes: employment. If there are other authors, they declare that they have no known competing financial interests or personal relationships that could have appeared to influence the work reported in this paper.
