# Supplementary material for: Awareness and perceptions of systemic inflammation in atherosclerotic cardiovascular disease and chronic kidney disease: The SPARK-CVD China survey
Source: Atheroscler Plus. 2026 Jun 6;65:100570. doi: 10.1016/j.athplu.2026.100570 (PMC13311795; doi:10.1016/j.athplu.2026.100570)
Supplement: Multimedia component 1 [file mmc1.pdf]

Supplementary Material

Awareness and perceptions of systemic inflammation in atherosclerotic cardiovascular disease and chronic kidney disease: The SPARK-CVD China survey

Supplementary Figure 1 Physicians' attitudes towards SI in patients with ASCVD and/or CKD

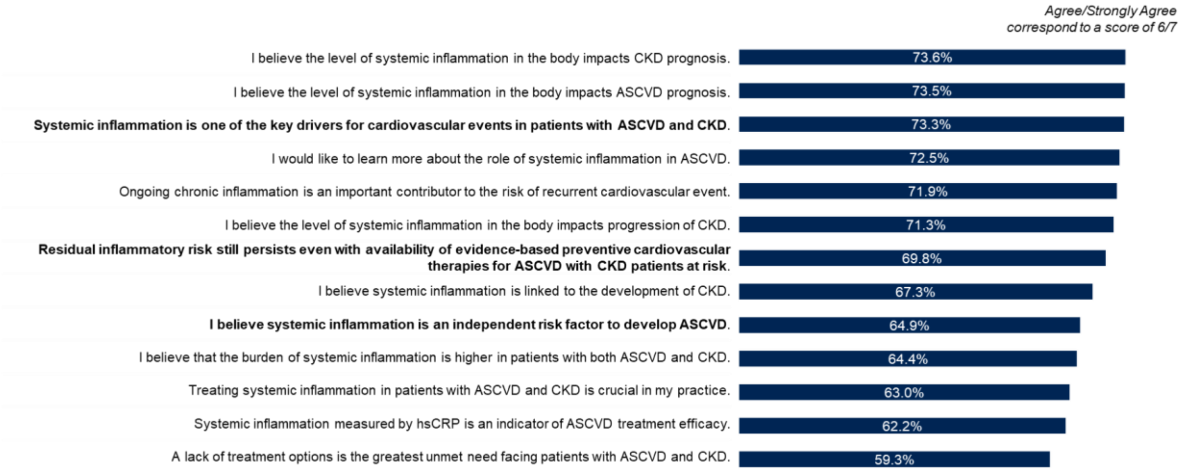

Attitudinal score based on a 7-point scale where 1 indicates "Strongly disagree" and 7 indicates "Strongly agree" (n = 1,500). Percentage of physicians who indicated "Agree" / "Strongly agree" (corresponding to 6/7 out of 7) were analysed.

Supplementary Figure 2 Unmet needs in the diagnosis and management of ASCVD and CKD

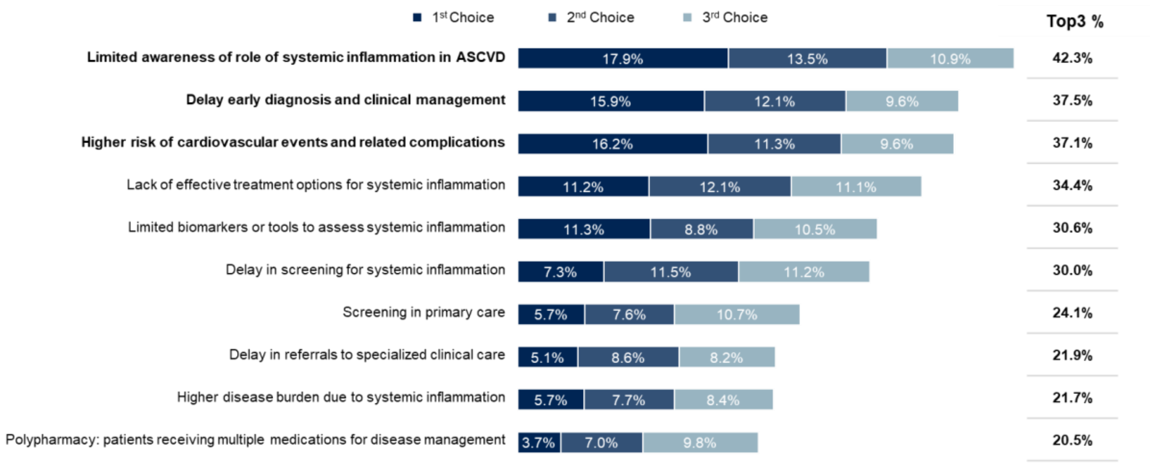

Top three unmet needs in the diagnosis and management of ASCVD and CKD (n = 1,500).

Supplementary Figure 3 Frequency of measuring/assessing SI in patients with ASCVD / ASCVD and CKD

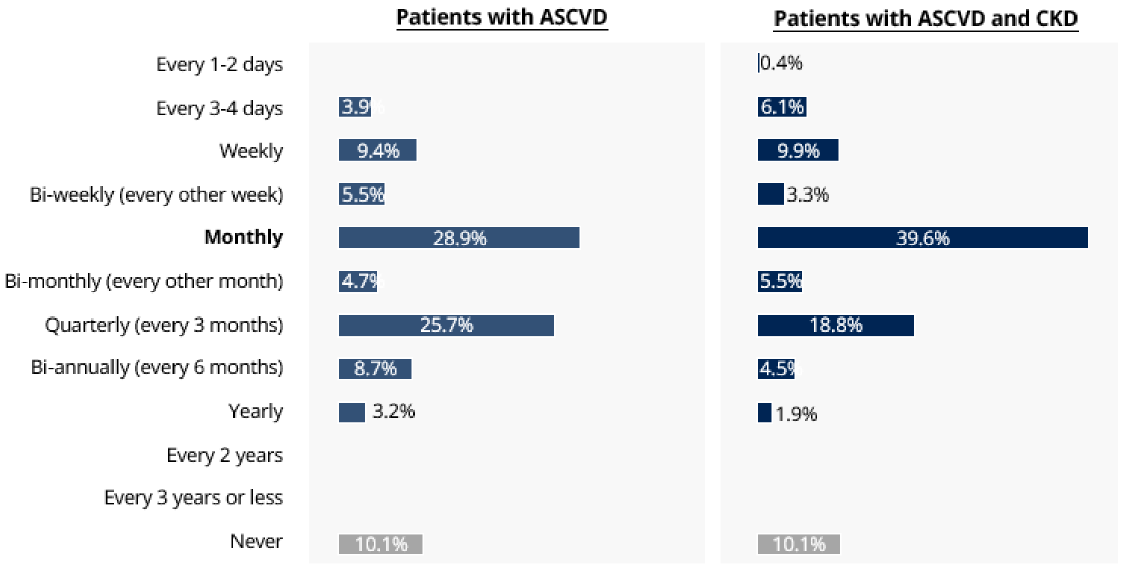

Frequency of measuring/assessing SI in patients with standalone ASCVD and in patients with both ASCVD and CKD by physicians (n = 1500).

Supplementary Figure 4 Minimum threshold range of hsCRP that impacts decision-making in managing ASCVD

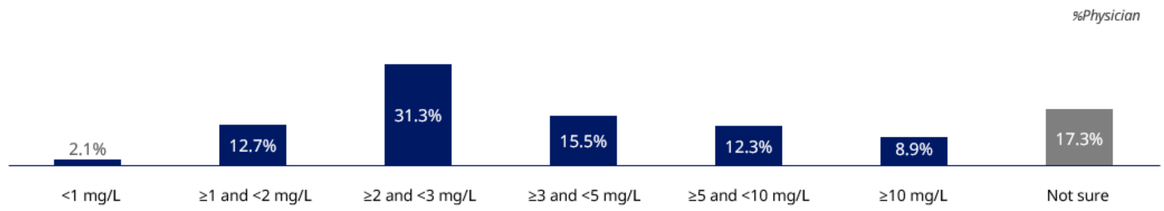

Single selection of the minimum threshold range of hsCRP from predefined list of choices (n = 1500).

Supplementary Figure 5 Importance and satisfaction rating on various outcomes in consideration of assessing SI in patients with ASCVD and CKD

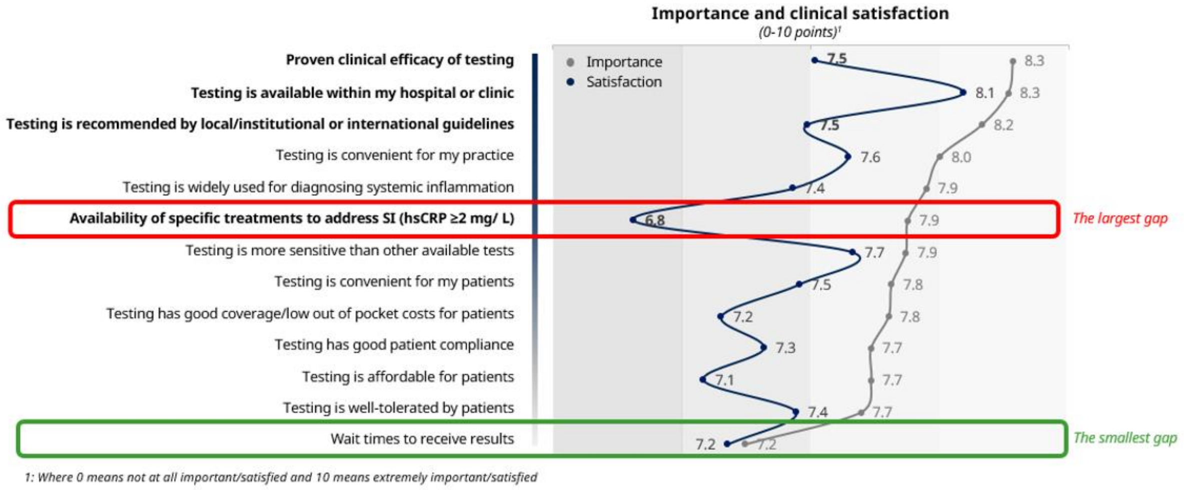

Importance and satisfaction score based on a 10-point scale, where 0 indicates "Not important at all" / "Not at all being met" and 10 indicates "extremely important" / "perfectly met" (n = 1,500).

Supplementary Figure 6 Hypothetical usage of colchicine and perception of GLP-1RA's anti-inflammatory effects

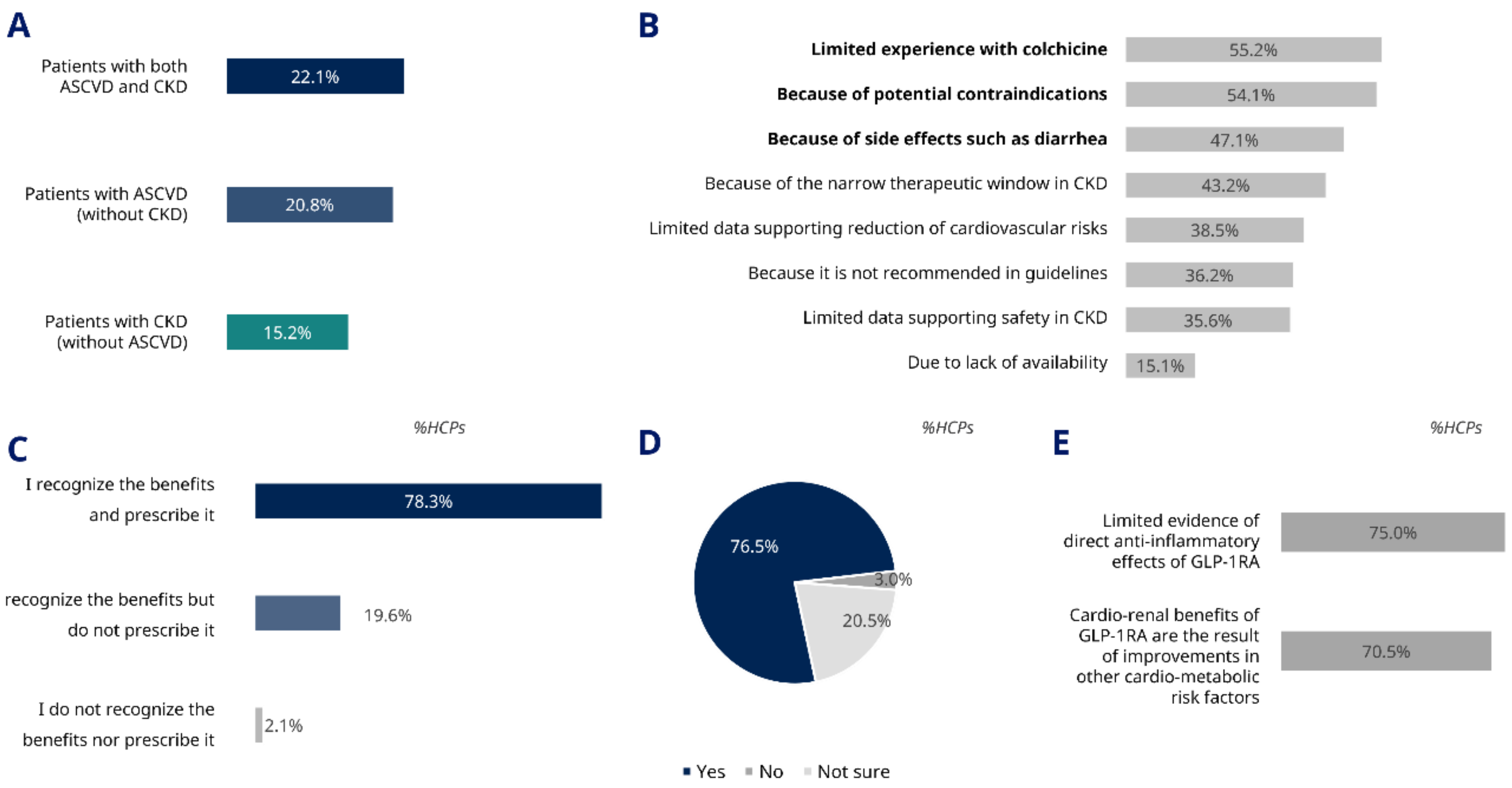

(A) Proportion of patients that would be prescribed with colchicine if hypothetically available for use (n = 1,500); (B) Reasons for not prescribing all CKD patients with colchicine (n = 1,500); (C) Recognition of GLP-1RA's cardiorenal benefits and prescription (n = 1,500); (D) Recognition

of GLP-1RA’s cardiorenal benefits being partially due to anti-inflammatory effects among physicians who recognised these benefits (n = 1,469); (E) Reasons for not recognising GLP-1RA’s anti-inflammatory effects among physicians who did not recognise them (n = 44).
